# Long-term Outcome of Neurological Complications after Infective Endocarditis

**DOI:** 10.1038/s41598-020-60995-3

**Published:** 2020-03-04

**Authors:** Ching-Chang Chen, Victor Chien-Chia Wu, Chien-Hung Chang, Chun-Ting Chen, Po-Chuan Hsieh, Zhuo-Hao Liu, Ho-Fai Wong, Chia-Hung Yang, An-Hsun Chou, Pao-Hsien Chu, Shao-Wei Chen

**Affiliations:** 1 Department of Neurosurgery, Linkou Chang Gung Memorial Hospital, Chang Gung University, Taoyuan City, Taiwan; 2 Department of Cardiology, Linkou Chang Gung Memorial Hospital, Chang Gung University, Taoyuan City, Taiwan; 3 Department of Neurology, Linkou Chang Gung Memorial Hospital & Chang Gung University, Taoyuan City, Taiwan; 4 Department of Radiology, Division of Neuroradiology, Linkou Chang Gung Memorial Hospital & Chang Gung University, Taoyuan City, Taiwan; 5 Department of Anesthesiology, Chang Gung Memorial Hospital, Linkou Medical Center, Chang Gung University, Taoyuan City, Taiwan; 6 Division of Thoracic and Cardiovascular Surgery, Department of Surgery, Linkou Chang Gung Memorial Hospital, Chang Gung University, Taoyuan City, Taiwan; 7 Center for Big Data Analytics and Statistics, Chang Gung Memorial Hospital, Linkou Medical Center, Taoyuan City, Taiwan

**Keywords:** Stroke, Cardiovascular diseases

## Abstract

Severe neurological complications following infective endocarditis remain a major problem with high mortality rate. The long-term neurological consequences following infective endocarditis remain uncertain. Otherwise, neurosurgeries could be performed after these complications; however, few clinical series have reported the results. Therefore, we utilized a large, nationwide database to unveil the long-term mortality and neurosurgical outcome following infective endocarditis. We included patients with a first-time discharge diagnosis of infective endocarditis between January 2001 and December 2013 during hospitalization. Patients were further divided into subgroups consisting of neurological complications under neurosurgical treatment and complications under non-neurosurgical treatment. Long-term result of symptomatic neurological complications after infective endocarditis and all-cause mortality after different kinds of neurosurgeries were analyzed. There were 16,495 patients with infective endocarditis included in this study. Symptomatic neurological complications occurred in 1,035 (6.27%) patients, of which 279 (26.96%) accepted neurosurgical procedures. Annual incidence of neurological complications gradually increased from 3.6% to 7.4% (*P* < 0.001). The mortality rate among these patients was higher than that among patients without complications (48.5% vs. 46.1%, *P* = 0.012, increased from 20% initially to nearly 50% over the 5-year follow-up). However, neurosurgery had no effect on the long-term mortality rate (50.9% vs. 47.6%, *P* = 0.451). Incidence of neurological complications post-infective endocarditis is increasing, and patients with these complications have higher mortality rates than patients without. Neurosurgery in these populations was not associated with higher long-term mortality. Therefore, it should not be ruled out as an option for those with neurological complications.

## Introduction

Neurological complications following infective endocarditis (IE) remain a major clinical problem with a poor prognosis^1–3^. Despite many changes in the epidemiology, diagnosis, and management of IE, the incidence of and morbidity associated with neurological complications have remained unchanged or even increased in recent years^4–6^. Neurological complications during an active course of IE have been reported to occur in 20–40% of patients^1–3,7,8^ and were associated with high mortality rates (22–58%)^1–3,9,10^. Neurological complications may refer to a broad spectrum of conditions ranging from nonspecific manifestations, such as encephalopathy, seizures or headaches, to fatal aneurysm rupture and cerebral hemorrhage^1–3^. In fact, nonspecific or transient symptoms show no obvious impact and relatively good outcomes^1^. However, the long-term outcome of symptomatic cerebral involvement within IE is still uncertain.

When these neurological complications occur, many neurosurgical procedures can be performed, depending on the diffident complications. Few clinical series have reported the impact of neurosurgical intervention upon neurological complications after IE and the long-term outcome following both valve surgery and neurosurgery in patients experiencing these complications. Therefore, our study utilized a large, nationwide database not only to identify long-term mortality associated with neurological complications of IE, but also to unveil an unprecedented dataset for neurosurgical outcomes after complications.

## Materials and Methods

### Data source

We conducted this nationwide cohort study using data from the National Health Insurance Research Database (NHIRD), which contains anonymous, secondary data released for only research purposes. The NHIRD is an administrative database of Taiwan’s National Health Insurance (NHI) program, which is a compulsory universal health insurance program covering nearly 100% of the 23.7 million residents of Taiwan^11,12^. The NHIRD contains comprehensive, health-related information such as diagnostic codes, medical procedures, images, and prescription details. The database uses information protection measures, including a strict data access procedure and encryption of all personal information with anonymous 32-digit identification numbers. Disease diagnoses are coded according to the *International Classification of Disease, Ninth Revision, Clinical Modification* (*ICD-9-CM*). The NHI program has a strict system of investigation and punishment. The diagnosis and insurance payment of IE should be based on Duke Criteria. The diagnosis of IE by the ICD-9-CM (code 421.0, 421.1, and 421.9) system and the NHI program is highly accurate and specific, as shown in many previous studies^12,13^. The study was exempted from full review by the Institutional Review Board of Chang Gung Memorial Hospital because of de-identified secondary data.

### Study design

**Figure 1:**
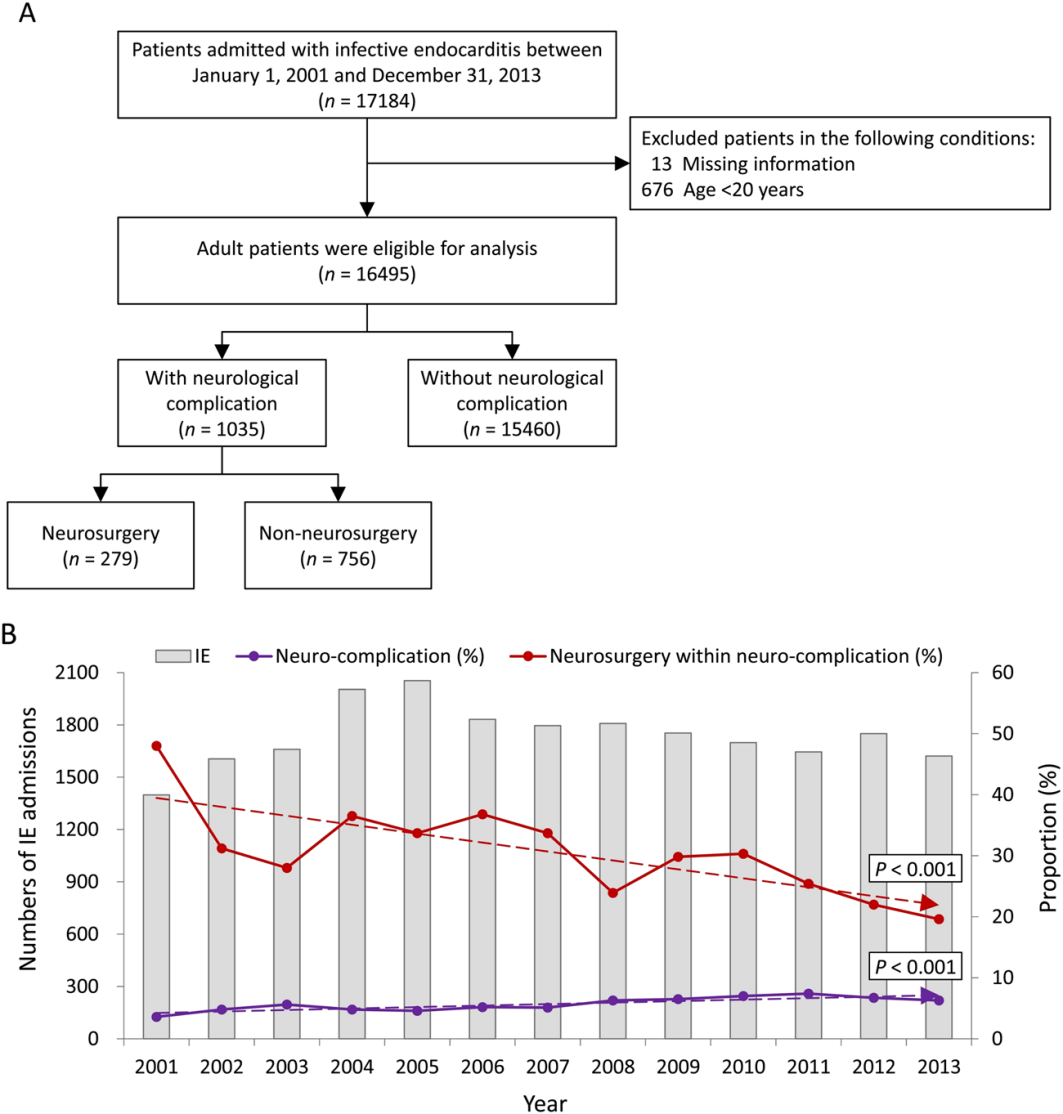
(**A**) Patient selection diagram. (**B**) Number of IE admissions, incidence of neurological complication, and proportion of those receiving neurosurgery across the study years. Abbreviation: IE, infective endocarditis.

This nationwide population-based, observational, retrospective cohort study was conducted to enroll the patients with a first-time discharge diagnosis of IE between January 2001 and December 2013 during hospitalization. Patients aged <20 years and with missing characteristics (i.e., sex and age) during follow-up were excluded. Figure 1A summarizes the inclusion and exclusion criteria in our study. The study patients were further divided into the IE with and without neurological complications. Neurological complications were defined as specific and definite intracerebral lesions, including ischemic and hemorrhagic strokes, cerebral aneurysms, and cerebral infection lesions, which occurred within 3 months during and after the admission for IE, using inpatient claim data. Non-specific or temporary conditions such as headache, vertigo, seizure, encephalopathy, or transient ischemic attack (TIA) were not included in this study. The clinical occurrence of IE with neurological complications was identified using ICD-9-CM codes 430–432 (hemorrhagic stroke) and 433, 434, and 436 (ischemic stroke), aneurysm with SAH (430) or unruptured cerebral aneurysm (437.3). Intracerebral infections were identified as cerebral abscess (324) and meningitis (437.4, 320–322). ICD-9-CM codes used for diagnosis are highly accurate and specific, as shown in many previous studies^12–20^. To improve the accuracy of diagnosis of the neurological complications, we double-checked the ICD-9-CM code concomitant with the procedure code of imaging studies for the brain, including computed tomography (CT) and magnetic resonance imaging (MRI), during the same admission^15,21^. Patients who accepted neurosurgical procedures were identified based on the ICD-9 CM codes and NHI reimbursement procedure codes^15,22,23^. Neurosurgical interventions for aneurysms included surgical clipping and endovascular coiling^14,23^. Neurosurgical procedures for intracranial hemorrhage (ICH), ischemic stroke, abscess, and meningitis are craniotomy to ICH, decompressive craniectomy or lobectomy for large brain infarction, craniotomy to remove brain mass, and intracranial pressure (ICP) monitor or external ventricular drain (EVD).

### Study outcomes

The primary outcomes of interest in this study were in-hospital mortality and all-cause mortality of IE with and without neurological complications in long-term follow-up. Secondary outcomes were in-hospital mortality and all-cause mortality of IE patients who had neurological complications with and without receiving neurosurgery. We aimed to further evaluate the effect of neurological complication on outcomes of IE with different severity. The aforementioned analyses were repeated in this cohort with severe IE, which was defined as receiving valve surgeries during the index admission. Mortality data were collected from the NHIRD when the patients withdrew from the NHI program^17^. The ICD-9 CM codes of identifying neurological complication, valve disease etiology, and comorbidities are listed in Supplemental Table 1. The blood culture positive rate was 22.3% in this study and the details of microbiology was shown in the Supplemental Figure.

### Statistical analysis

The trend of incidence of neurological complication and proportion of those receiving neurosurgery across the study years was tested using univariate logistic regression analysis, in which the study years (2001 to 2013) were treated as a continuous variable. Clinical and surgical characteristics of the patients between receiving neurosurgery or not were compared using the independent sample t-test for continuous variables or the chi-square test for categorical variables. The 5-year mortality rate in patients with and without neurological complications and in patients with neurological complication who did or did not receive neurosurgery, was compared using the log-rank test. Mortality rate in patients with neurological complication during various lengths of follow up by receiving neurosurgery or not in different types of neuro-complication was also compared using the log-rank test. Finally, patients with severe IE were identified and to compare the 5-year mortality rate in patients with and without neurological complication and in patients with neurological complication by receiving neurosurgery or not using the log-rank test. A two-sided *P* value < 0.05 was considered to be statistically significant and no adjustment of multiple testing (multiplicity) was made in this study. All statistical analyses were performed using SAS version 9.4 (SAS Institute, Cary, NC).

## Results

### Study population and incidence of IE with neurological complications

Between January 2001 and December 2013, a total of 16,495 patients were identified as having IE and included in our study (Fig. 1A). Definite neurological complications occurred in 1,035 (6.27%, 1,035 out of 16,495) patients. Among the patients who had neurological complications, 279 (26.96%, 279 out of 1035) patients accepted neurosurgical procedures for the complications and 756 patients maintained non-neurosurgical treatment. Figure 1B shows the annual incidence of neurological complications and neurosurgeries for complications from 2001 to 2013. Annual incidence of neurological complications of IE ranged from 3.6% to 7.4%. However, the incidence rose gradually year by year (*P* < 0.001). Patients who accepted neurosurgery for the complication decreased year by year (range from 48% to 19%, *P* < 0.001). Interestingly, after neurological complications, the rate of in-hospital mortality did not change over the most recent 10 years, whether in the neurosurgical group or not (Supplement Table 2).

### Neurosurgical and medical outcomes of IE with neurological complications

**Figure 2:**
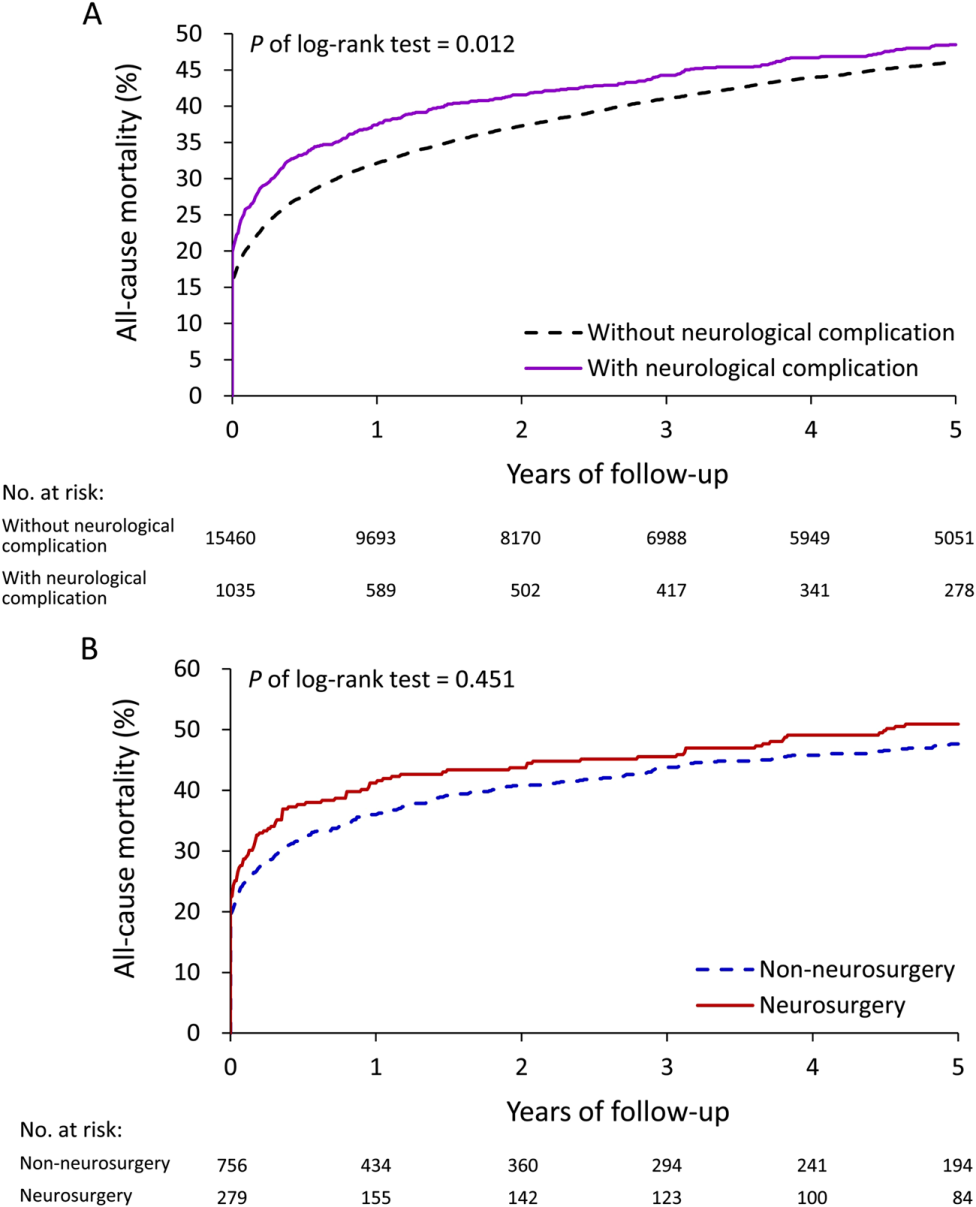
Unadjusted cumulative mortality rate in IE patients with and without neurological complication (**A**); patients with neurological complication with or without receiving neurosurgery (**B**).

Figure 2 shows the outcome of IE after a 5-year follow-up. The mortality rate of patients with neurological complications was higher than that of patients without complications (*P* = 0.012, Fig. 2A). The mortality rate of IE patients with neurological complications increased from about 20% initially to nearly 50% over the 5-year follow-up period. However, the differences between those with and without neurological complications reduced with time (48.5% vs. 46.1% at the fifth year, Fig. 2A).

When neurological complications occurred, some patients accepted medical treatment alone and some lesions required neurosurgical intervention. Table 1 reveals the clinical and surgical characteristics of the patients who did or did not receive neurosurgery after neurological complications. The non-neurosurgical subgroup was younger, had less comorbidities (including diabetes, hypertension, coronary artery disease, atrial fibrillation, and obstructive lung disease), and lower Charlson’s scores than the neurosurgery subgroup. The two groups significantly differed in the type of neurological complication, as brain aneurysms and intracerebral hemorrhage (ICH) were more common in the neurosurgery group whilst ischemic stroke was more common in the non-neurosurgery group. The prevalence of rheumatic heart disease and congenital heart disease was higher in the neurosurgery group (Table 1). During the 5-year follow-up period, no effect was observed on long-term mortality rate whether neurosurgery was performed or not (50.9% vs. 47.6%, *P* = 0.451, Fig. 2B).

**Table 1:** Clinical and surgical characteristics of the patients with neurological complication during the index IE admission by receiving neurosurgery or not.

Variable	Total (*n* = 1035)	Neurosurgery (*n* = 279)	Non-neurosurgery (*n* = 756)	*P* value
Age (years)	55.7 ± 17.1	48.3 ± 16.5	58.4 ± 16.5	<0.001
Age ≥65 years	337 (32.6)	45 (16.1)	292 (38.6)	<0.001
Male sex	693 (67.0)	200 (71.7)	493 (65.2)	0.049
Neurological complication				<0.001
Brain aneurysm	79 (7.6)	73 (26.2)	6 (0.8)	
Intracerebral hemorrhage	264 (25.5)	160 (57.3)	104 (13.8)	
Ischemic stroke	568 (54.9)	20 (7.2)	548 (72.5)	
Brain abscess	70 (6.8)	20 (7.2)	50 (6.6)	
Meningitis	54 (5.2)	6 (2.2)	48 (6.3)	
**Previous valve disease etiology**				
Rheumatic heart disease	56 (5.4)	26 (9.3)	30 (4.0)	0.001
Congenital heart disease	12 (1.2)	8 (2.9)	4 (0.5)	0.002
**Comorbid conditions**				
Previous stroke	185 (17.9)	44 (15.8)	141 (18.7)	0.283
Diabetes mellitus	248 (24.0)	49 (17.6)	199 (26.3)	0.003
Hypertension	362 (35.0)	65 (23.3)	297 (39.3)	<0.001
Heart failure	115 (11.1)	28 (10.0)	87 (11.5)	0.504
Coronary artery disease	174 (16.8)	34 (12.2)	140 (18.5)	0.016
Atrial fibrillation	91 (8.8)	15 (5.4)	76 (10.1)	0.018
Dialysis	63 (6.1)	11 (3.9)	52 (6.9)	0.080
Chronic obstructive pulmonary disease	60 (5.8)	8 (2.9)	52 (6.9)	0.014
Liver cirrhosis	73 (7.1)	14 (5.0)	59 (7.8)	0.120
Gastrointestinal bleeding	181 (17.5)	39 (14.0)	142 (18.8)	0.071
Drug abuse	28 (2.7)	13 (4.7)	15 (2.0)	0.019
Charlson’s total score	2.7 ± 2.4	2.4 ± 2.1	2.9 ± 2.5	0.002
Hospital level				0.746
Teaching hospital	602 (58.2)	160 (57.3)	442 (58.5)	
Regional/district hospital	433 (41.8)	119 (42.7)	314 (41.5)	
Hospital volume of IE between 2001 to 2013				0.416
1st quartile (1–168)	261 (25.2)	69 (24.7)	192 (25.4)	
2nd quartile (169–431)	269 (26.0)	79 (28.3)	190 (25.1)	
3rd quartile (448–703)	249 (24.1)	58 (20.8)	191 (25.3)	
4th quartile (745–1648)	256 (24.7)	73 (26.2)	183 (24.2)	
Severe IE (received valve surgery)	153 (14.8)	42 (15.1)	111 (14.7)	0.881
Follow up year	3.1 ± 3.5	3.3 ± 3.7	3.1 ± 3.4	0.253

**Figure 3:**
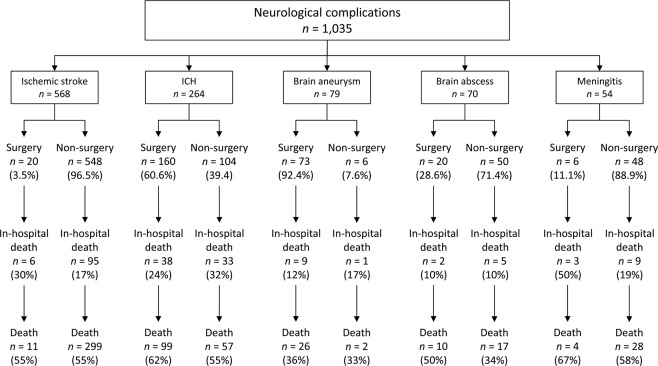
Therapeutic strategies and mortality according to the type of neurological complications. Abbreviation: ICH, intracerebral hemorrhage.

Figure 3 and Table 2 provide further therapeutic strategies and outcome according to different types of neurological complications. Ischemic stroke is the most common complication (54.9%, 568 out of 1,035). Most patients who suffered from ischemic stroke did not need neurosurgical treatment. However, the long-term mortality rate was still as high as 55%. On the other hand, most ICH (60.6%) needed neurosurgical intervention. The in-hospital and long-term neurosurgical mortality of IE with ICH was 24% and 62%. 92.4% of patients with intracerebral aneurysms were treated by craniotomy clipping or endovascular embolization. The surgical mortality was 12% in-hospital and 36% over a long time. Intracerebral abscess formation occurred in 6.8% (70 out of 1,035) of neurological complications. 28% of the abscesses that occurred were treated through neurosurgical removal. The surgical mortality rate was 10% in-hospital and 50% within the long-term follow up. Finally, meningitis (excluding abscess and aneurysm) is a rare complication and most (88.9%) of them need only medical treatment. But the long-term result is worst (58% in non-neurosurgical and 67% in neurosurgical subgroup).

**Table 2:** Mortality rate in patients with neurological complication during various length of follow up by receiving neurosurgery or not in different types of neurological complication.

Neurological complication	Length of follow up							
	1-year		3-year		5-year		At the end of follow-up	
	Neurosurgery	Non- neurosurgery	Neurosurgery	Non- neurosurgery	Neurosurgery	Non- neurosurgery	Neurosurgery	Non- neurosurgery
Ischemic stroke (*n* = 568)	9 (45.0)	198 (36.1)	10 (50.0)	247 (45.1)	10 (50.0)	270 (49.3)	11 (55.0)	299 (54.6)
ICH (*n* = 264)	77 (48.1)	46 (44.2)	86 (53.8)	51 (49.0)	96 (60.0)	52 (50.0)	99 (61.9)	57 (54.8)
Brain aneurysm (*n* = 79)	20 (27.4)	1 (16.7)	22 (30.1)	1 (16.7)	24 (32.9)	1 (16.7)	26 (35.6)	2 (33.3)
Brain abscess (*n* = 70)	6 (30.0)	10 (20.0)	6 (30.0)	12 (24.0)	8 (40.0)	14 (28.0)	10 (50.0)	17 (34.0)
Meningitis (*n* = 54)	3 (50.0)	17 (35.4)	3 (50.0)	20 (41.7)	4 (66.7)	23 (47.9)	4 (66.7)	28 (58.3)
Total (*n* = 1,035)	115 (41.2)	272 (36.0)	127 (45.5)	331 (43.8)	142 (50.9)	360 (47.6)	150 (53.8)	403 (53.3)

**Figure 4:**
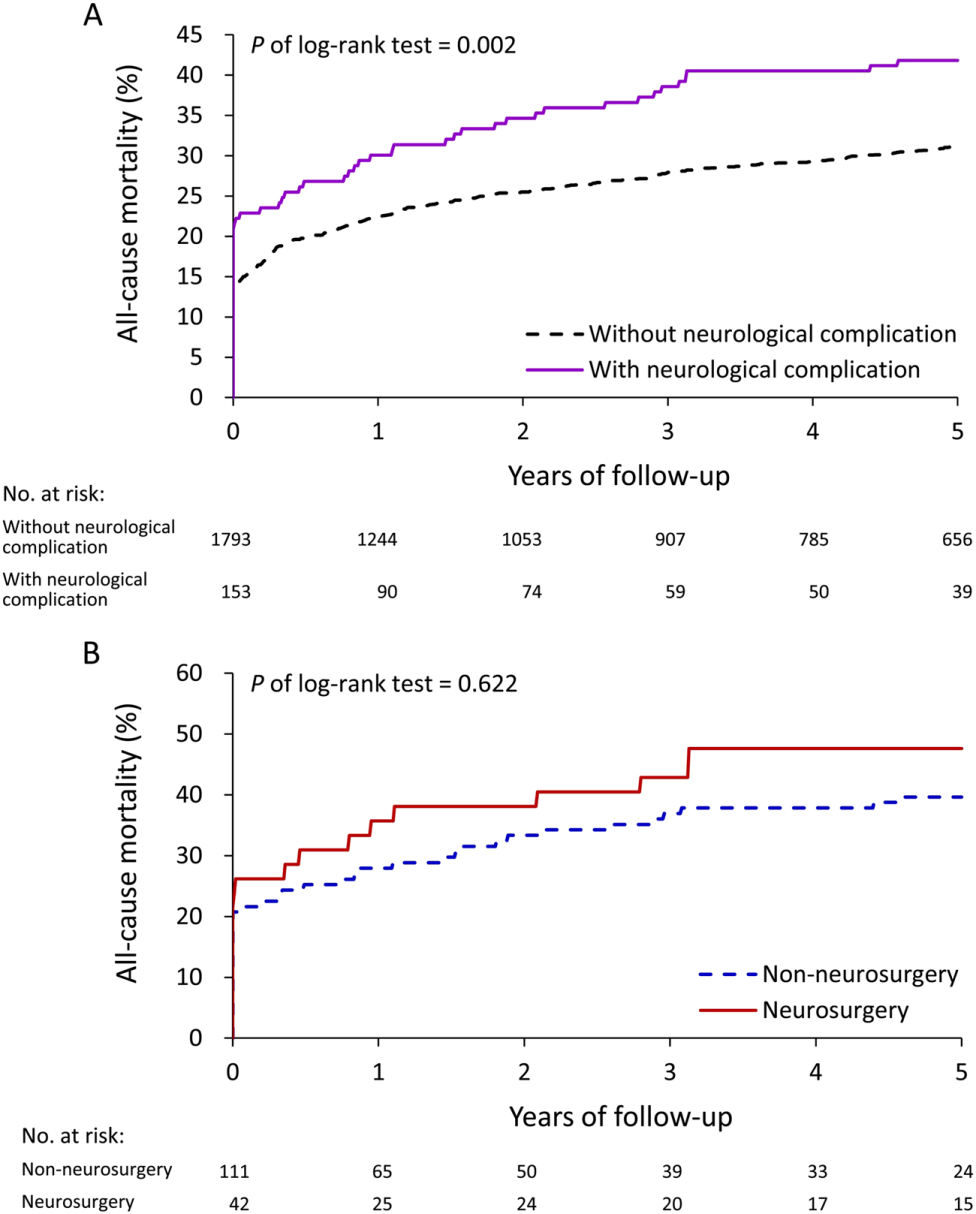
Unadjusted cumulative mortality rate in patients with severe IE (received valve surgery) by neurological complication (**A**); patients with severe IE (received valve surgery) and neurological complication by receiving neurosurgery or not (**B**).

Among the IE patients, valve surgeries were performed in 1946 patients. We defined these patients as having severe IE. Similarly, the patients with severe IE with neurological complications had higher mortality rate than those without complications (*P* = 0.002, Fig. 4A). During the 5 years follow-up, no effect was observed on long-term mortality rate in patients with severe IE irrespective of whether neurosurgery was performed or not (*P* = 0.622, Fig. 4B).

## Discussion

### Neurological complications of infective endocarditis

The overall frequency of neurological complications in the present cohort study was 6.27%. In the literature, the neurological complications during the active course of IE were reported to occur in 20–40% patients^1,3,7,8^. However, most of the studies include the asymptomatic cerebral lesions and non-specific or temporary conditions such as headache, vertigo, seizure, encephalopathy, or TIA, which showed no obvious impact and a relatively good outcome^1,24^. After exclusion of these minor lesions, moderate and severe symptomatic neurological complications occur in about 5–10%^1,3,25^, which was similar to that in our study. Over the past decade, we found the incidence of IE with neurological complications increased gradually year by year. Similar conditions were reported in the U.S, France, and England^4,6,26^. The result may be associated with an increase in invasive procedures leading to transient bacteremia^27^ and a gradual increase in high-risk populations that are older, diabetic, and on hemodialysis. In addition, there has been an increasing trend in the use of prosthetic cardiac devices^4,6,26^. Despite the increasing incidence of complications, patients who needed to accept neurosurgery for these complications decreased year by year. This may result from rapid diagnosis, early antibiotic treatment, and increasing application of imaging, such as CTA and MRA, for neurological survey before cardiac surgery^2,14,24^. The probabilities of neurological complications and recurrent stroke were known to rapidly decrease after early antimicrobial treatment^2,10,28^. The severity and progression of lesions that need neurosurgical interventions would thence reduce.

Typically, most of the neurological complications include ischemic stroke that is not fatal and does not require surgical intervention, regardless of the presence of symptoms. In our study, 54.88% (568 of 1035) of neurological complications involved ischemic stroke (Fig. 3) and only 3.5% needed surgical intervention. In the literature, IE-related brain abscess and meningitis were known but rarely reported neurological complications. In the limited reports, the incidence of a brain abscess may range from 2.8% to 30% based on the different diagnostic tools and criteria^29^. Endocarditis is an uncommon condition in bacterial meningitis, identified in 2%^30^ of patients and 1.1%^29^ complications of patients with IE. Our study revealed 0.7% cases of IE-related abscess and 0.5% cases of meningitis, confirmed by imaging.

### Outcome and impact of neurosurgery after neurological complications

Many previous studies showed that neurological complications are associated with increased mortality during IE, except non-specific complications^1,3,25^. Our study also revealed a higher mortality rate in patients with neurological complications than in patients without complications, whether they were in hospital or after 5-year follow-up. However, for patients undergoing neurosurgical intervention due to severe neurological complications, the long-term mortality rate was similar to that in the non-neurosurgical group. Otherwise, even for the patients in the subgroup of ICH and ischemic stroke, the long-term surgical and non-surgical results are still similar (as shown in Fig. 3 and Table 2). This result is in accordance with the results of a recent study by Thuny^1^ and Rutmman *et al*.^31^, who reported more improved survival in operated patients. Despite the fact that patients may have more comorbidities and increasingly severe neurologic status, neurosurgeons should not hesitate to perform operations under these conditions.

The indication and timing were still debated when severe IE that need cardiac surgery developed after the events of neurological complications. During the valve surgery, perioperative heparinization and hypotension potentially aggravate the neurological injury^32,33^. In many previous series^2,20,34–36^, cardiac surgery could be safely performed even after a recent ischemic neurological attack. On the other hand, a favorable postcardiac surgical result could also be achieved by postponing the operation in patients with a brain hemorrhage^2,35,37^. In our study, patients who had severe IE and underwent both cardiac and neurological operations also did not present with a poor long-term outcome. Collectively, these results demonstrate that cardiac and neurosurgeons should not hesitate to perform surgery when encountering cases of IE-associated neurological complications.

Usually, neurological complications are not fatal and can be temporary, or may gradually improve with medical treatment. However, when life-threatening situations such as large malignant infarction occur, the rates of disability and mortality are high^38^. Neurosurgical decompression can reduce the mortality rate from 50–80% to 30%^38^. The long-term result of our neurosurgical intervention for ischemic complications after IE seems similar as result for general events of large infarction. Most hemorrhagic stroke lesions also do not require and do not benefit from early surgical intervention^39^. However, patients with IE have a mortality rate of >50% from postoperative hemorrhage^40,41^. In our study, 60.6% of patients with IE needed surgical intervention, with 24% initial and 62% long-term mortality rate. Even without undergoing neurosurgery, the mortality risk was still higher than 50%, similar to that reported in the literature. The rupture rates for cerebral mycotic aneurysm due to IE are believed to be higher than those of non-infectious cerebral aneurysms with high mortality rate^42,43^. Reports generally recommend immediate intervention for ruptured and symptomatic aneurysms either by surgical or endovascular means^14,44^. The choice between surgical clip and endovascular technique depends on the lesion itself (numbers, size, and location), approachable parent and distal arteries, patient’s conditions, and priority of the medical institute^14,42,43^. A few previous clinical studies discussed the result of IE-related cerebral meningitis^29,30^. These scattered reports and our results both demonstrated that meningitis is a rare neurological complication but is associated with a high probability of an unfavorable outcome^30^. The reason may be destructive neurological dysfunction by diffused brain infection^30^. The result of brain abscess has not been reported previously. Our study was the first to reveal mortality rates including 10% in-hospital, 50% long-term neurosurgical, and 34% non-surgical mortality.

### Limitations

This study has some limitations due to the administrative nature of the NHIRD. First, this is an observational study and has the biases associated with retrospective database study. Despite making allowances for known confounders by using statistic adjustment, we could investigate associations but we could not infer causation. Residual confounding may have occurred due to unmeasured or unknown confounders. In addition, the diagnosis of neurological complications was strictly based on the ICD-9-CM system annexed with brain CT or MRI. The registry data did not include the location and size of the stroke. Otherwise, the severity of each stroke and each patient’s functional status remains unknown and cannot be compared. The lack of available cardiac echo results (such as vegetation size and ejection fraction) might have a possible effect on the outcomes measured. Finally, in our study, the database does not contain the exact mean time between neurosurgeries and the neurological complications that occurred, which may influence the result. Despite these limitations, our study included a large population with diverse groups of patients who underwent long-term follow-up at various hospitals. Furthermore, the NHIRD enabled near-complete follow-up with little missing data through linkage to national mortality records. Therefore, we believe that these findings have broad generalizability and provide a valuable guide to the long-term treatment.

## Conclusion

Patients with neurological complications after IE have a higher mortality rate than non-complicated patients. In the past years, despite the advance of medical treatment and diagnostic tools, the incidence of neurological complications after IE remains increasing. However, neurosurgery in these populations did not associate with a higher mortality. Neurosurgeons should not hesitate to operate when considering the IE-associated neurological complications. Aggressive intervention may be a lifesaving procedure, with acceptable long-term outcomes in patients regarded as unlikely to survive or survive with prominent neurologic deficits.

## Supplementary information

Supplementary information

Supplementary information 2

Supplementary information 3
