# Supplementary material for: Long-term Outcome of Neurological Complications after Infective Endocarditis
Source: Sci Rep. 2020 Mar 4;10:3994. doi: 10.1038/s41598-020-60995-3 (PMC7055329; doi:10.1038/s41598-020-60995-3)

Long-term Outcome of Neurological Complications after Infective Endocarditis

Ching-Chang Chen, MD[a]; Victor Chien-Chia Wu, MD[b]; Chien-Hung Chang, MD[c]; Chun-Ting Chen, MD[a]; Po-Chuan Hsieh, MD[a]; Zhuo-Hao Liu, MD, PhD[a] ; Ho-Fai Wong, MD[d]; Chia-Hung Yang, MD[b] ; An-Hsun Chou MD, PhD[e]; Pao-Hsien Chu, MD[b]; Shao-Wei Chen, MD, PhD[f,g]

[a] Department of Neurosurgery, Linkou Chang Gung Memorial Hospital, Chang Gung University, Taoyuan City, Taiwan

[b] Department of Cardiology, Linkou Chang Gung Memorial Hospital, Chang Gung University, Taoyuan City, Taiwan

[c] Department of Neurology, Linkou Chang Gung Memorial Hospital & Chang Gung University, Taoyuan City, Taiwan

[d] Department of Radiology, Division of Neuroradiology, Linkou Chang Gung Memorial Hospital & Chang Gung University, Taoyuan City, Taiwan

[e] Department of Anesthesiology, Chang Gung Memorial Hospital, Linkou Medical Center, Chang Gung University, Taoyuan City, Taiwan

[f] Division of Thoracic and Cardiovascular Surgery, Department of Surgery, Linkou Chang Gung Memorial Hospital, Chang Gung University, Taoyuan City, Taiwan

[g] Center for Big Data Analytics and Statistics, Chang Gung Memorial Hospital, Linkou Medical Center, Taoyuan City, Taiwan

Supplementary Figure： Microbiology of Infective Endocarditis

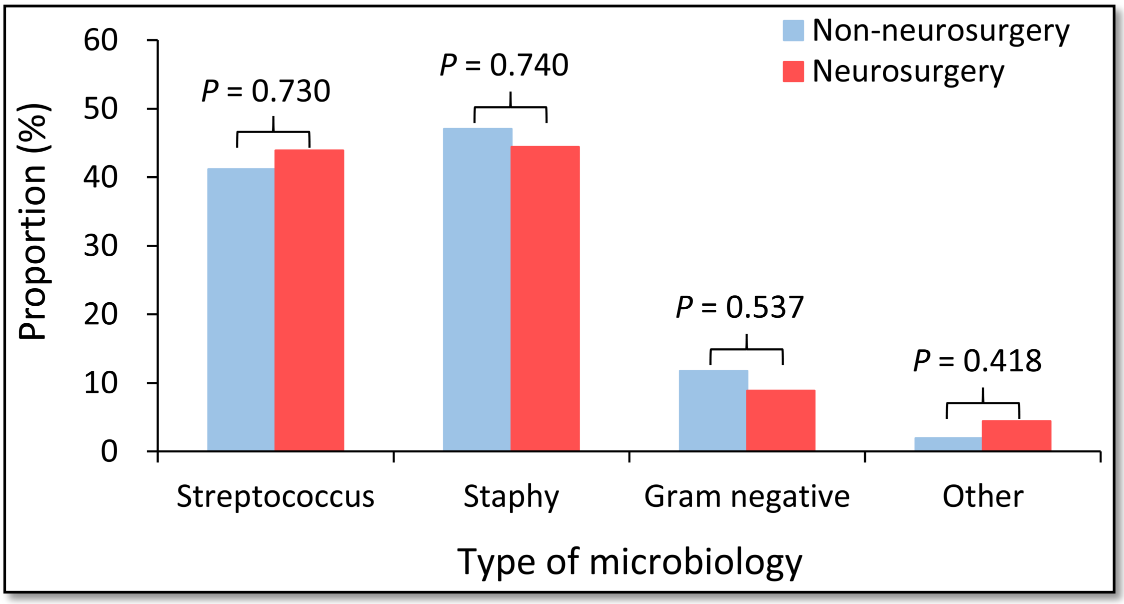

Supplement: Supplementary file 1 — Supplementary information [file 41598_2020_60995_MOESM1_ESM.pdf]
