# Supplementary material for: Long-term Outcome of Neurological Complications after Infective Endocarditis
Source: Sci Rep. 2020 Mar 4;10:3994. doi: 10.1038/s41598-020-60995-3 (PMC7055329; doi:10.1038/s41598-020-60995-3)
Supplement: Supplementary file 2 — Supplementary information 2 [file 41598_2020_60995_MOESM2_ESM.pdf]

Long-term Outcome of Neurological Complications after Infective Endocarditis

Ching-Chang Chen, MD[a]; Victor Chien-Chia Wu, MD[b]; Chien-Hung Chang, MD[c]; Chun-Ting Chen, MD[a]; Po-Chuan Hsieh, MD[a]; Zhuo-Hao Liu, MD, PhD[a] ; Ho-Fai Wong, MD[d]; Chia-Hung Yang, MD[b] ; An-Hsun Chou MD, PhD[e]; Pao-Hsien Chu, MD[b]; Shao-Wei Chen, MD, PhD[f,g]

[a] Department of Neurosurgery, Linkou Chang Gung Memorial Hospital, Chang Gung University, Taoyuan City, Taiwan

[b] Department of Cardiology, Linkou Chang Gung Memorial Hospital, Chang Gung University, Taoyuan City, Taiwan

[c] Department of Neurology, Linkou Chang Gung Memorial Hospital & Chang Gung University, Taoyuan City, Taiwan

[d] Department of Radiology, Division of Neuroradiology, Linkou Chang Gung Memorial Hospital & Chang Gung University, Taoyuan City, Taiwan

[e] Department of Anesthesiology, Chang Gung Memorial Hospital, Linkou Medical Center, Chang Gung University, Taoyuan City, Taiwan

[f] Division of Thoracic and Cardiovascular Surgery, Department of Surgery, Linkou Chang Gung Memorial Hospital, Chang Gung University, Taoyuan City, Taiwan

[g] Center for Big Data Analytics and Statistics, Chang Gung Memorial Hospital, Linkou Medical Center, Taoyuan City, Taiwan

Supplemental Table 1. ICD-9-CM code used in the current study

Variable	ICD-9-CM code
Infective endocarditis (IE)	421.0, 421.1, and 421.9
Neurological complication	
Ischemic stroke	433.xx, 434.xx, and 436.xx
Hemorrhagic stroke	430.xx–432.xx
Brain aneurysm	aneurysm with subarachnoid hemorrhage (430) or un-ruptured cerebral aneurysm (437.3)
Brain abscess	324
Meningitis	437.4, 320.xx-322.xx
Rheumatic heart disease	394.0, 394.1, 394.2, 395.xx, 398.9x
Congenital heart disease	745.xx, 746.xx
Stroke	430.xx–432.xx, 433.xx, 434.xx, and 436.xx
Diabetes mellitus	250.xx
Hypertension	401.xx–405.xx
Heart failure	428.xx
Coronary artery disease	410.xx-414.xx
Atrial fibrillation	427.31
Dialysis	585 (Catastrophic illness card)
Chronic obstructive pulmonary disease	491.xx, 492.xx, 496.xx
Liver cirrhosis	571.5, 571.6, 571.2
Gastrointestinal bleeding	530.21, 530.7, 530.82, 531.xx-534.xx,535.xx, 537.83, 537.84, 578.xx
Drug abuse	304.xx, 305.xx

ICD-9-CM, International Classification of Diseases, Ninth Revision, Clinical Modification.
