# Supplementary material for: Long-term Outcome of Neurological Complications after Infective Endocarditis
Source: Sci Rep. 2020 Mar 4;10:3994. doi: 10.1038/s41598-020-60995-3 (PMC7055329; doi:10.1038/s41598-020-60995-3)
Supplement: Supplementary file 3 — Supplementary information 3 [file 41598_2020_60995_MOESM3_ESM.pdf]

Long-term Outcome of Neurological Complications after Infective Endocarditis

Ching-Chang Chen, MD[a]; Victor Chien-Chia Wu, MD[b]; Chien-Hung Chang, MD[c]; Chun-Ting Chen, MD[a]; Po-Chuan Hsieh, MD[a]; Zhuo-Hao Liu, MD, PhD[a] ; Ho-Fai Wong, MD[d]; Chia-Hung Yang, MD[b] ; An-Hsun Chou MD, PhD[e]; Pao-Hsien Chu, MD[b]; Shao-Wei Chen, MD, PhD[f,g]

[a] Department of Neurosurgery, Linkou Chang Gung Memorial Hospital, Chang Gung University, Taoyuan City, Taiwan

[b] Department of Cardiology, Linkou Chang Gung Memorial Hospital, Chang Gung University, Taoyuan City, Taiwan

[c] Department of Neurology, Linkou Chang Gung Memorial Hospital & Chang Gung University, Taoyuan City, Taiwan

[d] Department of Radiology, Division of Neuroradiology, Linkou Chang Gung Memorial Hospital & Chang Gung University, Taoyuan City, Taiwan

[e] Department of Anesthesiology, Chang Gung Memorial Hospital, Linkou Medical Center, Chang Gung University, Taoyuan City, Taiwan

[f] Division of Thoracic and Cardiovascular Surgery, Department of Surgery, Linkou Chang Gung Memorial Hospital, Chang Gung University, Taoyuan City, Taiwan

[g] Center for Big Data Analytics and Statistics, Chang Gung Memorial Hospital, Linkou Medical Center, Taoyuan City, Taiwan

Supplemental Table 2. In-hospital mortality rate in the medical and neurosurgical groups after neurological complications

Index year	Total (*n* = 1,286)	Non-neurosurgery (*n* = 905)	Neurosurgery (*n* = 381)
2001	4 (8.0)	1 (3.9)	3 (12.5)
2002	15 (19.5)	11 (20.8)	4 (16.7)
2003	14 (15.1)	10 (14.9)	4 (15.4)
2004	14 (14.6)	9 (14.8)	5 (14.3)
2005	17 (17.9)	10 (15.9)	7 (21.9)
2006	25 (26.3)	14 (23.3)	11 (31.4)
2007	16 (17.4)	13 (21.3)	3 (9.7)
2008	24 (21.2)	18 (20.9)	6 (22.2)
2009	20 (17.5)	13 (16.3)	7 (20.6)
2010	16 (13.5)	11 (13.3)	5 (13.9)
2011	23 (18.9)	13 (14.3)	10 (32.3)
2012	29 (24.6)	21 (22.8)	8 (30.8)
2013	21 (20.6)	18 (22.0)	3 (15.0)
Total	238(18.5)	162(17.9)	76(19.9)
P of trend	0.532	0.270	0.167
